# Predicting chemical bioavailability using microarray gene expression data and regression modeling: A tale of three explosive compounds

**DOI:** 10.1186/s12864-016-2541-5

**Published:** 2016-03-08

**Authors:** Ping Gong, Xiaofei Nan, Natalie D. Barker, Robert E. Boyd, Yixin Chen, Dawn E. Wilkins, David R. Johnson, Burton C. Suedel, Edward J. Perkins

**Affiliations:** Environmental Laboratory, US Army Engineer Research and Development Center, Vicksburg, MS 39180 USA; Department of Computer and Information Science, University of Mississippi, Oxford, Mississippi 38677 USA; Bennett Aerospace Inc., Cary, North Carolina 27518 USA; GHD, Dallas, Texas 75234 USA; Present Address: School of Information Engineering, Zhengzhou University, Zhengzhou, Henan 450001 China

**Keywords:** Tissue residue, Global gene expression profiling, Predictor genes, Predictive regression modeling, TNT (2,4,6-Trinitrotoluene), RDX (1,3,5-Trinitro-1,3,5-triazacyclohexane), HMX (Octahydro-1,3,5,7-tetranitro-1,3,5,7-tetrazocine), Earthworm (*Eisenia fetida*)

## Abstract

**Background:**

Chemical bioavailability is an important dose metric in environmental risk assessment. Although many approaches have been used to evaluate bioavailability, not a single approach is free from limitations. Previously, we developed a new genomics-based approach that integrated microarray technology and regression modeling for predicting bioavailability (tissue residue) of explosives compounds in exposed earthworms. In the present study, we further compared 18 different regression models and performed variable selection simultaneously with parameter estimation.

**Results:**

This refined approach was applied to both previously collected and newly acquired earthworm microarray gene expression datasets for three explosive compounds. Our results demonstrate that a prediction accuracy of R^2^ = 0.71–0.82 was achievable at a relatively low model complexity with as few as 3–10 predictor genes per model. These results are much more encouraging than our previous ones.

**Conclusion:**

This study has demonstrated that our approach is promising for bioavailability measurement, which warrants further studies of mixed contamination scenarios in field settings

**Electronic supplementary material:**

The online version of this article (doi:10.1186/s12864-016-2541-5) contains supplementary material, which is available to authorized users.

## Background

Bioavailability processes were defined in a 2003 National Research Council (NRC) report [1] as the individual physical, chemical, and biological interactions that determine the exposure of plants and animals to chemicals associated with soils and sediments. In environmental risk assessment, the amount of chemicals taken up by an animal or plant is termed dose, or, interchangeably tissue residue, body burden or chemical bioavailability. A distinction exists between dose and exposure as the latter is defined as the amount of chemicals present in the immediate environment where the organism is exposed to. Soils are a major sink for many environmental contaminants including explosives compounds. Soil contamination by military unique compounds is a serious environmental concern that can result in the formation of chemical residues in tissue of exposed organisms [2]. Since many site-specific biotic and abiotic factors can modify the form, mobility and availability of these contaminants, the actual exposure risk to ecological receptors may be less than that suggested by their total concentration. Therefore, the extent to which chemicals are bio-available has significant implications for risk management and remedial decision-making at contaminated sites.

A myriad of biological, physical and chemical approaches have been used to evaluate bioavailability of chemicals [3]. For instance, biological approaches include techniques measuring organismal uptake (body burden or bioaccumulation), response, and toxicity. Tissue residues or body burden of chemicals, often determined using analytical chemistry methods, can represent the bio-available or biologically effective concentration at the target site. However, little consensus exists about optimal approaches for measuring bioavailability. As summarized in the aforementioned NRC report [1] and a recent review [3], no single tool is free from limitations and none can be applied universally. An intensive effort to develop mechanistic tools or models based on mechanisms is critical to future development of bioavailability tools [1, 3].

Recent advances have prompted the application of genomics-based technologies to ecological risk assessment, including screening, tiered testing, monitoring, remediation, and regulatory decision-making (see [4, 5] for details). Toxicogenomics has been extensively applied to assess toxicological effects, especially in biomarker discovery and toxicity mechanistic investigations. On the other hand, toxicogenomics also possesses a great potential for providing a quantitative measure of chemical exposure. Nevertheless, the application of toxicogenomics to exposure assessment has been relatively under-explored. Previously, we applied microarray technology to profile gene expression in earthworms exposed to explosives 2,4,6-trinitrotoluene (TNT, CAS Number 118-96-7) or 1,3,5-trinitro-1,3,5-triazacyclohexane (RDX, CAS Number 121-82-4) for 4 or 14 days, and built multivariate regression models to quantitatively predict earthworm tissue residue of these two compounds [2]. The models, however, showed only a modest predictive power, explaining close to half of the variance for TNT tissue residue and one-quarter of the variance for RDX tissue residue.

The present study was motivated to improve the quantitative predictive power of regression modeling based on genome-wide gene expression data. Our hypothesis was that small sets of predictor genes could be identified and used to build multivariate regression models for quantitative prediction of tissue residue levels of explosives compounds. Consistent with our previous study, the overall goal of this study was to investigate the feasibility of using gene expression data to assess animal exposure. To achieve this goal, we re-analyzed the previous datasets by separating the 4-day exposure from the 14-day exposure, and expanding from two regression methods to 18 methods. In addition, we have also generated a new dataset from HMX-exposed earthworms (HMX: octahydro-1,3,5,7-tetranitro-1,3,5,7-tetrazocine, CAS Number 2691-41-0) and applied the same approach to analyzing it. Our results demonstrated that much higher prediction accuracies were attained indicating that microarray gene expression coupled with multivariate regression modeling is a viable approach for assessing chemical bioavailability.

## Methods

**Fig. 1:**
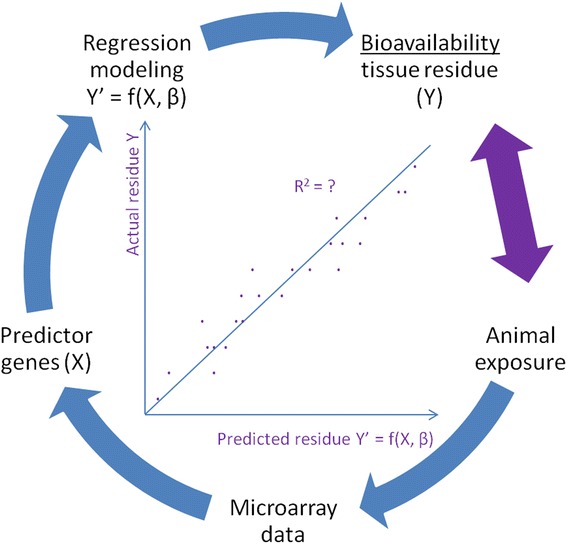
The overall experimental approach. See the Methods section for explanation

The overall experimental approach is depicted in Fig. 1. We first exposed earthworms to explosives compounds and measured tissue residues (*y*) and gene expression profiles in both exposed earthworms and unexposed controls. Then we identified small sets of predictor genes (*x*) to build regression models (*y’* = *f*(*x*,β), where *y’* is the predicted residue and β is a coefficient vector. These models were applied to predict the bioavailability (tissue residues) in exposed worms using only predictor gene expression data. Finally, the actual measured tissue residues (*y*) were compared with the predicted values (*y’*) to determine prediction accuracy evaluated by correlation coefficients (R^2^). Our ultimate goal was to apply these prediction models to estimate the unknown bioavailability in worms from their gene expression data (as indicated by the purple double-headed arrow).

### Experimental design

Previously, we collected a large 248-sample dataset with measurements of transcriptome-wide gene expression [6] and tissue residue of two explosives chemicals, [U-^14^C]-labeled TNT and RDX, in the earthworm *Eisenia fetida* (see details in [2]). Briefly, three sets of experiments were conducted by exposing mature adult worms bearing a clear clitellum in a pristine sandy loam soil amended with TNT or RDX. Nominal exposure concentrations were as follows: 0, 6, 12, 24, 48, 96 mg [U-^14^C] TNT/kg, or 8, 16, 32, 64, 128 mg [U-^14^C] RDX/kg for 4 days (the 1st set) or 14 days (the 3rd set). The 4-day exposure was repeated (the 2nd set) with the same TNT concentrations but different RDX concentrations (2, 4, 8, 16 and 32 mg/kg).

Following the same experimental design, we exposed adult worms to [U-^14^C]-labeled HMX for 4, 14 and 28 days at nominal concentrations of 0 (blank control and solvent control), 8, 16, 32, 64 and 128 mg/kg in the same silt loam soil as in our previous study [6]. [U-^14^C]HMX was purchased from DuPont NEN (Boston, MA) with an initial specific activity of 86.4 mCi/mmol. Earthworms were reared in-house in a continuous lab culture as previously described [7]. Ten worms were exposed in 250 g (dry weight) of soil per each treatment. The nominal soil concentrations were verified to possess less than 10 % variations from target concentrations using a High Performance Liquid Chromatography equipped with a Radioactivity Flow Detector (HPLC-RFD) [2]. Upon termination, all worms were flash-frozen in liquid nitrogen, stored at−80 °C, lyophilized at −40 °C, and homogenized.

### Tissue residue analysis for HMX

All ten worms per treatment were analyzed for tissue residue. For tissue HMX-residue analysis, triplicate subsamples (10–15 mg each) were digested in 1 mL of 0.6 N ScintiGest Tissue Solubilizer (Fisher Scientific) for 18 h [8]. Radioactivity was determined by adding 200 μl of tissue digest to Ultima Gold scintillation cocktail (Perkin-Elmer, Waltham, MA) using a Packard TriCarb 2500TR Liquid Scintillation Counter (Meriden, CT). Prior to the analysis, we validated this method (see more details in [8]) by comparing it with our previously used methods. Testing results showed that the solubilization method yielded 92 % ± 3 % (mean ± standard error, *n* = 19) of the radioactivity measured previously by using oxidization methods for the same TNT- or RDX-exposed worms [2].

### Gene expression profiling for HMX-exposed worms

Total RNA was extracted from five of the ten worms per treatment using an RNeasy mini kit (Qiagen, Valencia, CA). Each RNA sample was hybridized to the custom-designed Agilent 15 K *E. fetida* oligo array (AMADID#021219; Santa Clara, CA) previously used for generating the TNT and RDX microarray datasets [6]. This array contained 15208 unique transcript-targeted 60-mer oligo probes (8 arrays per slide). Details of array hybridization, gene expression data acquisition and pre-processing were described elsewhere [2, 6].

### Identification of differentially expressed (DE) genes

DE genes were identified for all three datasets (TNT, RDX and HMX) among multiple concentrations using a multivariate permutation random-variance *t*-test (two-class) or *F*-test (multiple-class) implemented in BRB-ArrayTools version 4.2.1 [9, 10]. A gene was considered statistically significant if it achieved 80 % confidence that the false discovery rate (FDR) was less than 10 %. DE genes were derived for individual exposure duration and explosives compound separately.

### Regression prediction modeling

For residue prediction, we chose 18 different multivariate regression models and employed double-looped, 10-fold cross-validation as described in Statnikov et al. [11] to assess prediction accuracy. The inner loop was used to determine the optimal value of parameters (in a cross-validated fashion) for training in the outer loop. Model performance (prediction accuracy) was estimated in the outer loop by training on all splits but one, and using the remaining one for testing. Coefficient of determination (R^2^) was calculated using Pearson’s formula to describe prediction accuracy or “goodness of fit”, i.e., how well the tissue residue predicted by a regression model represented the actually measured residue of a worm tissue sample. The 18 models include six linear models (Multivariate, Robust, Ridge, LASSO regularization, Elastic net regularization, and Support Vector Regression (SVR)) and 12 nonlinear models (Stepwise, Ridge Polynomial, Ridge Exponential, Ridge Gaussian kernel, SVR Polynomial, SVR Gaussian kernel, SVR Sigmoid kernel, Nadaraya-Watson kernel, Inverse regression, Loglog, Regression tree, and Random Forest) (see Additional file 1 for model description and references). Matlab codes were scripted to implement array data preprocessing, regression, and cross-validation, and are available upon request.

## Results

### Selection of predictor genes from TNT and RDX gene expression datasets

We reanalyzed the TNT and RDX microarray datasets by separating the 4-day sample set from the 14-day sample set. Differentially expressed (DE) genes were identified among different treatments (classes) of 4-day or 14-day earthworm samples using a multivariate permutation test [9]. Statistical reanalysis of the 14-day exposure gene expression data resulted in six and three DE genes for TNT and RDX, respectively (Additional file 2 and Additional file 3). For the 4-day TNT exposure, 1758, 886 and 4985 genes were inferred as DE genes from the original exposure (containing six classes), the repeat exposure (four classes) and the original vs. repeat controls (two classes), respectively (Additional file 2). A group of 118 DE genes were found to be common between the original and the repeat TNT exposures. This group was further reduced to 53 genes after excluding genes also found to be significantly altered between the original and the repeat controls (see worksheet “OriginalD4” in Additional file 2). Similarly, 488 and 2682 DE genes were derived from the original RDX and the repeat RDX exposures (six classes each), respectively, with 178 genes in common (Additional file 3). Twenty-six genes out of the 178-gene group remained after excluding the same DE genes appearing in the controls comparison (TNT and RDX exposures shared the control treatments; see worksheet “RepeatD4” in Additional file 3).

The final sets of identified DE genes are shown in Additional file 2 and Additional file 3. The low numbers of DE genes found in 14-day exposures are consistent with our previous report [6]. We obtained more DE genes than previously for the 4-day exposures because of the reduced statistics stringency (80 % confidence level and 10 % FDR vs. 99 % confidence level and ten false positive genes [6]). However, worms used in the original and the repeat exposures exhibited significant differences that were reflected as nearly 1/3 of all 15 K profiled genes differentially expressed between the two control groups (see worksheet “D4controls” in Additional file 2 and Additional file 3). Therefore, we chose to remove these genes not responding specifically to 4-day TNT or RDX exposure from the final DE gene lists (see worksheet “D4_finalDEgenes-expression” in Additional file 2 and Additional file 3).

### Regression predictive modeling for TNT and RDX tissue residue

Using the aforesaid final sets of DE genes as predictor genes, the 18 regression methods displayed varied power in predicting worm tissue residues of TNT and RDX (Table 1). The predictive power was assessed using the coefficient of determination (R^2^) as a measure of the accuracy of the data model. No single method was placed as the best performer for all four datasets. For instance, the multivariate linear regression model was the best performer for the 14-day TNT exposure dataset, but its performance was relatively weak on the 4-day TNT exposure dataset. Quite a few models such as LASSO, Elastic net, the Ridge family models, the SVR family models except for SVR Sigmoid, and Nadaraya-Watson performed consistently well across all four datasets. In contrast, some models like SVR Sigmoid, Loglog, and reverse regression performed poorly or even appeared inapplicable to the datasets.

**Table 1:** Performance of 18 regression modeling methods on four datasets assessed by coefficient of determination (R^2^, mean ± standard deviation, *n* = 10) estimated from ten runs of 10-fold cross-validation with values of the best performing method for each dataset shown in bold

Regression method	RDX_D4	RDX_D14	TNT_D4	TNT_D14
Predictor size (gene #)	26	3	53	6
Linear				
Multivariate	0.62 ± 0.19	0.65 ± 0.12	0.42 ± 0.14	**0.72 ± 0.18**
Robust	0.63 ± 0.14	0.65 ± 0.13	NA	0.67 ± 0.15
Ridge	0.65 ± 0.15	0.65 ± 0.13	0.73 ± 0.15	0.71 ± 0.16
LASSO	0.65 ± 0.18	0.65 ± 0.14	0.73 ± 0.15	0.69 ± 0.15
Elastic net	**0.66 ± 0.20**	0.66 ± 0.13	0.75 ± 0.19	0.69 ± 0.17
SVR	0.60 ± 0.15	0.68 ± 0.14	0.74 ± 0.16	0.66 ± 0.16
Nonlinear				
Stepwise	0.42 ± 0.21	0.69 ± 0.14	0.33 ± 0.21	0.6 ± 0.16
Ridge Polynomial	0.62 ± 0.18	**0.71 ± 0.12**	0.71 ± 0.14	0.66 ± 0.16
Ridge Exponential	0.65 ± 0.13	0.67 ± 0.13	0.68 ± 0.14	0.67 ± 0.17
Ridge Gaussian	0.64 ± 0.14	0.70 ± 0.15	0.43 ± 0.13	0.64 ± 0.16
SVR Polynomial	0.61 ± 0.15	0.68 ± 0.14	0.70 ± 0.12	0.63 ± 0.16
SVR Gaussian	0.63 ± 0.13	0.68 ± 0.14	0.74 ± 0.12	0.67 ± 0.13
SVR Sigmoid	0.17 ± 0.00	NA	0.08 ± 0.00	NA
Nadaraya-Watson	0.54 ± 0.09	0.68 ± 0.16	0.73 ± 0.17	0.67 ± 0.13
Inverse	0.44 ± 0.14	NA	0.31 ± 0.10	NA
Loglog	NA	NA	NA	NA
Regression Tree	0.53 ± 0.10	0.59 ± 0.13	0.73 ± 0.12	0.54 ± 0.14
Random Forest	0.60 ± 0.12	0.59 ± 0.16	**0.75 ± 0.10**	0.70 ± 0.17

*RDX_D4* 4-day RDX exposure, *RDX_D14* 14-day RDX exposure, *TNT_D4* 4-day TNT exposure, *TNT_D14* 14-day TNT exposure, *NA* not available. See Additional file 5 for the lists and annotation of predictor genes

**Fig. 2:**
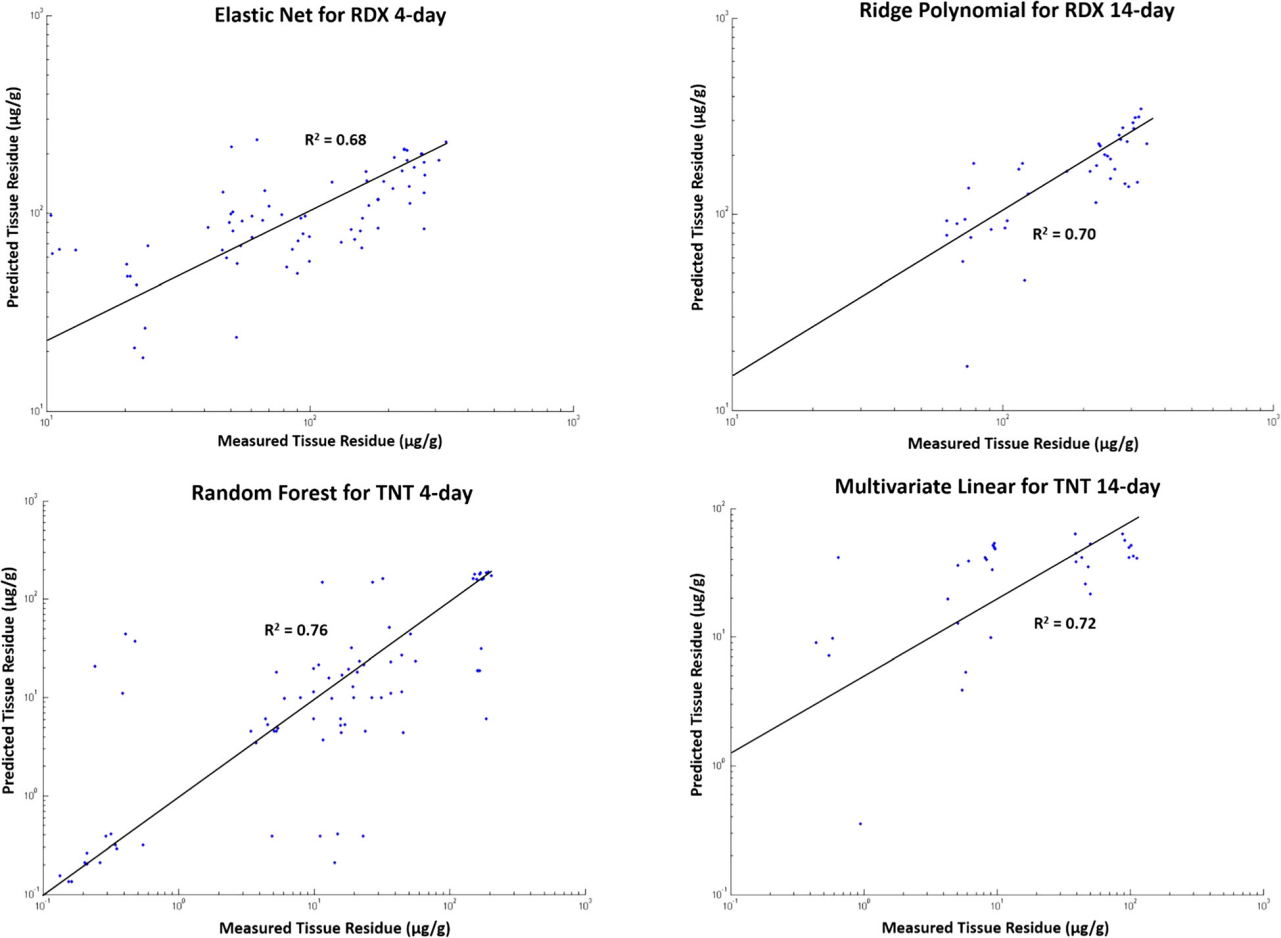
The average predicted versus the measured tissue residues of TNT or RDX in all 4-day or 14-day exposed samples using their respective best performing models

The predictive power of the best performers was remarkably improved in comparison to our previously published results [2]. On average, these models explained 75 % (TNT) or 66 % (RDX) variance of the 4-day samples, and 72 % (TNT) or 71 % (RDX) variance of the 14-day samples (Fig. 2). In our previous study, the best prediction models explained roughly one-quarter and less than one-half of the variance for RDX and TNT, respectively [2].

### Tissue residue in HMX-exposed earthworms

**Fig. 3:**
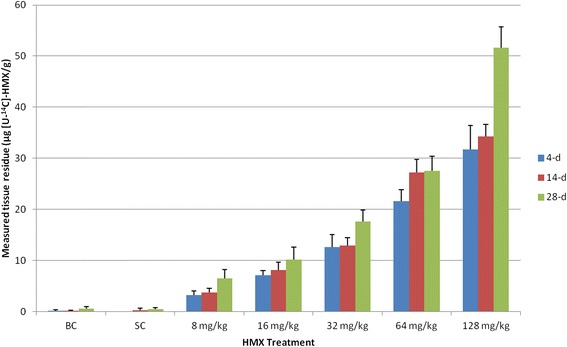
Tissue residue of radio-labeled HMX measured in earthworms exposed for 4-, 14-, and 28-days (see Supplementary file 3 for raw data). Data are represented as mean (column) + standard deviation (error bar) with *n* = 10. BC = blank control; SC = solvent control

The measured tissue residue of radio-labeled HMX increased with the increasing nominal amendment concentration and also with the duration of exposure (Fig. 3 and Additional file 4 worksheet “Residue”). Trace amounts of HMX were detected in some of the two control groups, which are equivalent to the background noise level or the lower detection limit of the analytical method. The worm tissue residue did not appear to plateau at the highest amendment level, suggesting that more HMX could be taken up from soil by the earthworm, given a higher amendment concentration.

### Predictor genes selection for HMX gene expression dataset

The HMX microarray gene expression dataset consists of three exposure groups (4-, 14- and 28-day) of 40 arrays, i.e., 8 treatments (including *T* = 0 sampling, solvent control, blank control, and five HMX concentrations) × 5 replicates. The two control groups in all three exposures showed no significant difference because no DE gene was identifiable at the same settings as above. So, the two groups were combined as one control group for subsequent statistical analyses. The three Day 0 samplings (3-class comparison) exhibited little difference with only seven derived DE genes, suggesting that worms used in all three exposures were nearly identical and that animal batches had a minimal impact on the test results (see worksheet “T0” in Additional file 4). Only 2, 2, and six genes were inferred as DE genes for the 4-, 14-, and 28-day exposures, respectively (see worksheets “4D”, “14D” and “28D” in Additional file 4, respectively). There was no overlap among all four sets of inferred DE genes (Day 0, 4, 14 and 28, see worksheet “Predictor genes” in Additional file 4). Using DE genes as predictors, regression modeling resulted in a modest predictive power for the 4-day (two genes) and 14-day (two genes) exposures with the best performer only explaining an average of 55 % variance (see worksheet “Performance” in Additional file 4). Performance of the 6 predictor genes on the 28-day dataset was much better with the highest R^2^ of 0.75. All these preliminary results are provided in Additional file 4.

In order to improve the predictive power, a different approach was used to identify additional predictor genes. We ran a correlation test to determine the degree of dependence between each expressed gene and the tissue residue. Then, all 15 K genes were ranked according to their coefficients of correlation (*r*). With a cutoff of |*r*| = 0.6, we obtained 21, 12 and 29 most correlated genes for the 4-d, 14-d and 28-d exposures, respectively, which were considered potential predictor genes (see worksheet “Predictor genes” in Additional file 4). Genes at the top of the most correlated genes lists were most positively correlated with tissue residue, while those at the bottom most negatively correlated with tissue residue. Some of the genes on these lists were also DE genes. We conducted performance analyses of the incremental new sets of predictor genes by adding two or three genes from the top or the bottom of the most correlated genes list at a time to the DE genes to form a new predictor gene set. Results (not shown) indicate that addition of the top two and the bottom two had an optimal enhancement in prediction accuracy. Therefore, the final revised sets of predictor genes consisted of DE genes, the top two and the bottom two genes on the most correlated genes lists.

### Prediction outcome for HMX gene expression dataset

**Fig. 4:**
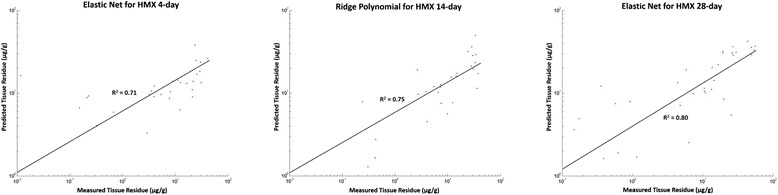
Prediction results of 4-, 14- and 28-day HMX-exposed earthworm tissue residues using the best performing models (shown are the results of a single run of 10-fold cross-validation)

Using the revised sets of predictor genes (see worksheet “Predictor genes” in Additional file 4), the regression models achieved substantial enhancement in prediction accuracy (Table 2 and worksheet “Performance” in Additional file 4). Particularly, prediction outcomes for the 4- and 14-day exposures improved by nearly 20 % as the best performers for the two datasets both explained 72–73 % of the variance (Fig. 4). The 28-day dataset did not improve as much (7 % increase) largely because five out of the six DE genes (|*r*| > 0.6) were among the most correlated genes list with one (TA1-161768) being the most negatively correlated gene (*r* = −0.738) and another (TA2-167546) the third most positively correlated gene (*r* = 0.674) (worksheet “Predictor genes” in Additional file 4). In contrast, none of the four DE genes derived from the 4- and 14-day datasets were on the most correlated genes lists. Models that performed well on the TNT and RDX datasets also did consistently well on the HMX datasets. Three models, SVR Sigmoid, inverse regression and loglog regression, were not suitable for all datasets (Tables 1 and 2), probably because of transformation and normalization operations in data pre-processing.

**Table 2:** Performance of 18 regression modeling methods on the three HMX exposure datasets assessed by coefficient of determination (R^2^, mean ± standard deviation, *n* = 10) estimated from ten runs of 10-fold cross-validation with values of the best performing method shown in bold

Regression method	D4	D14	D28
Predictor size (gene #)	6	6	10
Linear			
Multivariate	0.53 ± 0.15	0.52 ± 0.15	0.58 ± 0.15
Robust	0.66 ± 0.12	0.72 ± 0.09	0.79 ± 0.02
Ridge	0.67 ± 0.10	0.70 ± 0.11	0.81 ± 0.02
LASSO	0.69 ± 0.10	0.72 ± 0.10	0.81 ± 0.04
Elastic net	**0.72 ± 0.09**	0.71 ± 0.11	**0.82 ± 0.03**
SVR	0.70 ± 0.10	0.65 ± 0.09	0.81 ± 0.05
Nonlinear			
Stepwise	0.67 ± 0.07	0.66 ± 0.11	0.79 ± 0.05
Ridge Polynomial	0.63 ± 0.11	**0.73 ± 0.08**	0.76 ± 0.05
Ridge Exponential	0.68 ± 0.08	0.68 ± 0.09	0.79 ± 0.04
Ridge Gaussian	0.51 ± 0.16	0.56 ± 0.14	0.66 ± 0.06
SVR Polynomial	0.69 ± 0.11	0.64 ± 0.11	0.79 ± 0.06
SVR Gaussian	0.65 ± 0.09	0.60 ± 0.10	0.73 ± 0.10
SVR Sigmoid	0.48 ± 0.15	0.49 ± 0.15	0.68 ± 0.12
Nadaraya-Watson	0.68 ± 0.09	0.67 ± 0.09	0.80 ± 0.04
Inverse	NA	NA	NA
Loglog	NA	NA	NA
Regression Tree	0.56 ± 0.15	0.61 ± 0.14	0.65 ± 0.13
Random Forest	0.55 ± 0.16	0.60 ± 0.13	0.69 ± 0.10

*D4* 4-day HMX exposure, D14 14-day HMX exposure, *D28* 28-day HMX exposure, *NA* not available. See Additional file 5 for the lists and annotation of predictor genes

## Discussion

Quantitative prediction of a phenotype or trait using high dimensional gene expression data has been long explored in many research fields such as human diseases [12, 13], animal breeding [14] and plant genetics [14, 15]. The phenotypes of prediction interest are broad and have included thrombocytosis etiologies [16], acute myeloid leukemia resistance [17] and breast cancer tumor response [18] to chemotherapy in biomedical research, cattle milk yield in animal breeding [14], and wheat grain yield [14] and plant pathogen infection severity [15] in plant genetics. The methodology of predictive modeling varies widely from Bayesian network-based approaches [19] to Bayesian hierarchical regression modeling [15] and ordinary multivariate regression [18]. The reported prediction accuracy also varies from one study to another, implying significant challenges and opportunities co-existing in this field [13].

Similar to the aforementioned fields of research, we have also sought to use transcriptomic data coupled with regression modeling to predict a phenotype, i.e., chemical residues in animals [2, 20]. Although prediction accuracies were unsatisfactory in our previous study, we have significantly improved them in the present study without adding to the complexity of regression models. The size of predictor gene sets has been reduced to three to ten genes for all datasets except the 4-day TNT and RDX exposures (Tables 1 and 2), which are likely the lowest number of predictors possible [16, 18], given the complicated processes involved in residue formation. Although there is still room for further improvement, the accuracies obtained here are comparable to those reported in other quantitative trait prediction studies [14–19].

It has been reported that variable or feature selection is a key step towards accurate prediction [2, 15]. To select features (genes) that have high predictive power as predictors, many different strategies have been developed, including *t*- or *F*-test based approaches to identify differentially expressed genes (e.g., [17]), correlation based methods to rank genes (e.g., [18]), machine learning based techniques (e.g., [21, 22]), and some combinations of the three types of strategies (e.g., [2, 6, 15]). In the present study, we have used both *F*-test and correlation based approaches to select for subsets of predictor genes. A variety of regression models were applied to describe relationships between the response variable (i.e., tissue residue) and a set of predictors through a regression function involving some parameter(s) to be estimated from data [13]. Given the time-dependent nature of tissue residue (response variable) and gene expression levels (predictors) [20], the datasets were split by exposure duration. Results indicate that this step remarkably improved the prediction outcome for both TNT and RDX exposures. To be used as predictors, altered levels of a given transcript need not be connected to a specific biologic end point, nor do the specific functions of all the mRNA molecules have to be known, although such information would be valuable [20]. Bioinformatic annotation of all 110 selected earthworm predictor genes using Blast2GO [23] shows that only 47 of them (43 %) have meaningful biological functions and that their contribution to residue formation remain largely unknown (Additional file 5).

Recently, there has been an intense interest in performing variable selection simultaneously with parameter estimation in predictive modeling. Frequently applied approaches include Bayesian methods (e.g., Gibbs Variable Selection (GVS), Stochastic Search Variable Selection (SSVS), adaptive shrinkage with Jeffreys’ prior or a Laplacian prior, and reversible jump Markov Chain Monte Carlo (MCMC)) [24] and LASSO, Ridge or Elastic net regularization methods [25, 26]. Given such a large variety, there is no consensus with regard to what method is the universally best performer. For instance, O’Hara and Sillanpaa [24] tested several Bayesian variable selection methods on both simulated and experimentally collected data and concluded that SSVS, reversible jump MCMC and adaptive shrinkage methods all worked well, but the choice of which method was better depended on the priors that were used, and also on how they were implemented. Similarly, Fu et al. [22] concluded that SVR, partial least squares regression and multiple linear regression yielded higher prediction accuracies for one dataset but transcriptome-based distances worked better on another dataset. In the present study, we also observed that LASSO, Ridge and Elastic net regularization methods performed almost equally well on all datasets, whereas inverse and loglog regression methods performed poorly on the datasets in a consistent fashion.

It is worth noting that the approach employed in this study faces several challenges when applied to residue prediction. First, compared with other existing approaches, it requires a more extensive effort to identify and optimize a set of predictor genes. Second, if the species of interest does not have a transcriptome-wide microarray available, one has to either design the microarray from scratch or use the array of a closely related species (e.g., using *E. fetida*-specific array for *E. andrei* [27]), which may limit its applicability. Third, as a novel approach, there is no doubt that it is still in its embryonic phase and that its full potential and limitations are not yet explored thoroughly. For instance, our approach may be applied to quantitative predictions in drug discovery such as predictive ranking of new drug toxicity and/or potency as they share similarities in high data dimensionality and transcriptomic profiling based on either microarray- or RNA-Seq/next-generation sequencing-based data.

## Conclusions

Chemical residue in exposed animals is an important dose metric in environmental risk assessment. The formation of tissue residue involves a number of complex biological processes which can be reflected as expression profiles in microarray experiments. Building predictive models of tissue residue based on gene expression would help to accurately assess how much chemical an animal has been exposed to thereby enabling assessment of bio-available toxicant levels in the environment. It is our belief that the microarray technology coupled with regression modeling provides an innovative and promising tool towards this direction. The natural next steps are to demonstrate the applicability and prediction power of this new approach in scenarios of contaminants mixtures and also at field contaminated military sites. Ultimately, information collected from such studies will be used to support further development of predictive modeling for toxicogenomic measures of exposure [20].

### Availability of supporting data

The microarray datasets were deposited in the National Center for Biotechnology Information (NCBI)’s Gene Expression Omnibus (GEO) database as series GSE42866 (HMX dataset; http://www.ncbi.nlm.nih.gov/geo/query/acc.cgi?acc=GSE42866) and GSE18495 (TNT and RDX datasets; http://www.ncbi.nlm.nih.gov/geo/query/acc.cgi?acc=GSE18495). The custom-designed earthworm (*Eisenia fetida*) microarray with 15 K oligo probes is accessible as GEO platform GPL9420.

## Additional files

Additional file 1: Description of 18 regression models and their references. (DOCX 21 kb) Additional file 2: Identified DE genes (predictor genes), their expression data and measured tissue residue results for 4- and 14-day TNT-exposed earthworms. (XLSX 996 kb) Additional file 3: Identified DE genes (predictor genes), their expression data and measured tissue residue results for 4- and 14-day RDX-exposed earthworms. (XLSX 1109 kb) Additional file 4: Identified DE genes and other selected predictor genes, measured tissue residue of HMX, and the performance of 18 regression models using only DE genes as predictors for the 4-, 14- and 28-day exposures. (XLSX 51 kb) Additional file 5: Functional annotation of 110 identified predictor genes for TNT, RDX and HMX residues using Blast2GO.(XLSX 29 kb)

## Abbreviations

DE: Differentially expressed
FDR: False detection rate
GEO: Gene expression omnibus
GVS: Gibbs variable selection
HMX: High melting eXplosive (octahydro-1,3,5,7-tetranitro-1,3,5,7-tetrazocine)
HPLC-RFD: High performance liquid chromatography equipped with a radioactivity flow detector
MCMC: Markov Chain Monte Carlo
NCBI: National Center for Biotechnology Information
NRC: National Research Council
RDX: Royal Department formula X (1,3,5-trinitro-1,3,5-triazacyclohexane)
SSVS: Stochastic search variable selection
SVR: Support vector regression
TNT: 2,4,6-TriNitroToluene

## References

[1] National Research Council (US) Committee on Bioavailability of Contaminants in Soils and Sediments: Bioavailability of Contaminants in Soils and Sediments: Processes, Tools, and Applications. Washington DC: The National Academies Press; 2003.

[2] Gong P, Loh PR, Barker ND, Tucker G, Wang N, Zhang C (2012). Building quantitative prediction models for tissue residue of two explosives compounds in earthworms from microarray gene expression data. Environ Sci Technol.

[3] Katayama A, Bhula R, Burns GR, Carazo E, Felsot A, Hamilton D (2010). Bioavailability of xenobiotics in the soil environment. Rev Environ Contam Toxicol.

[4] Ankley GT, Miracle AL, Perkins EJ, Daston GP (2008). Genomics in regulatory ecotoxicology: applications and challenges.

[5] US Environmental Protection Agency (2004). Potential implications of genomics for regulatory and risk assessment applications at EPA. EPA 100/B-04/002.

[6] Li Y, Wang N, Perkins EJ, Zhang C, Gong P (2010). Identification and optimization of classifier genes from multi-class earthworm microarray dataset. PLoS One.

[7] Pirooznia M, Gong P, Guan X, Inouye LS, Yang K, Perkins EJ (2007). Cloning, analysis and functional annotation of expressed sequence tags from the earthworm *Eisenia fetida*. BMC Bioinf.

[8] Belden JB, Lotufo GR, Chambliss CK, Fisher JC, Johnson DR, Boyd RE (2011). Accumulation of 14C-trinitrotoluene and related nonextractable (bound) residues in Eisenia fetida. Environ Pollut.

[9] Korn EL, Li MC, McShane LM, Simon R (2007). An investigation of two multivariate permutation methods for controlling the false discovery proportion. Stat Med.

[10] Simon RM, Korn EL, McShane LM, Radmacher MD, Wright GW, Zhao Y (2003). Design and analysis of DNA microarray investigations.

[11] Statnikov A, Aliferis CF, Tsamardinos I, Hardin D, Levy S (2005). A comprehensive evaluation of multicategory classification methods for microarray gene expression cancer diagnosis. Bioinformatics.

[12] Lee SH, van der Werf JH, Hayes BJ, Goddard ME, Visscher PM (2008). Predicting unobserved phenotypes for complex traits from whole-genome SNP data. PLoS Genet.

[13] delos Campos G, Gianola D, Allison DB (2010). Predicting genetic predisposition in humans: the promise of whole-genome markers. Nat Rev Genet.

[14] Long N, Gianola D, Rosa GJ, Weigel KA (2011). Application of support vector regression to genome-assisted prediction of quantitative traits. Theor Appl Genet.

[15] Bhattacharjee M, Sillanpaa MJ (2011). A bayesian mixed regression based prediction of quantitative traits from molecular marker and gene expression data. PLoS One.

[16] Gnatenko DV, Zhu W, Xu X, Samuel ET, Monaghan M, Zarrabi MH (2010). Class prediction models of thrombocytosis using genetic biomarkers. Blood.

[17] Heuser M, Wingen LU, Steinemann D, Cario G, von NN, Tauscher M (2005). Gene-expression profiles and their association with drug resistance in adult acute myeloid leukemia. Haematologica.

[18] Sano H, Wada S, Eguchi H, Osaki A, Saeki T, Nishiyama M (2012). Quantitative prediction of tumor response to neoadjuvant chemotherapy in breast cancer: novel marker genes and prediction model using the expression levels. Breast Cancer.

[19] Chang HH, McGeachie M (2011). Phenotype prediction by integrative network analysis of SNP and gene expression microarrays. Conf Proc IEEE Eng Med Biol Soc.

[20] National Research Council (US) Committee on Applications of Toxicogenomic Technologies to Predictive Toxicology (2007). Applications of toxicogenomic technologies to predictive toxicology and risk assessment.

[21] Liu Q, Sung AH, Chen Z, Liu J, Chen L, Qiao M (2011). Gene selection and classification for cancer microarray data based on machine learning and similarity measures. BMC Genomics.

[22] Fu J, Falke KC, Thiemann A, Schrag TA, Melchinger AE, Scholten S (2012). Partial least squares regression, support vector machine regression, and transcriptome-based distances for prediction of maize hybrid performance with gene expression data. Theor Appl Genet.

[23] Conesa A, Gotz S, Garcia-Gomez JM, Terol J, Talon M, Robles M (2005). Blast2GO: a universal tool for annotation, visualization and analysis in functional genomics research. Bioinformatics.

[24] O’Hara RB, Sillanpaa LJ (2009). A review of Bayesian variable selection methods: what, how and which. Bayesian Anal.

[25] delos Campos G, Naya H, Gianola D, Crossa J, Legarra A, Manfredi E (2009). Predicting quantitative traits with regression models for dense molecular markers and pedigree. Genetics.

[26] Wu TT, Chen YF, Hastie T, Sobel E, Lange K (2009). Genome-wide association analysis by lasso penalized logistic regression. Bioinformatics.

[27] van Ommen Kloeke AE, Gong P, Ellers J, Roelofs D (2014). Effects of a natural toxin on life history and gene expression of *Eisenia andrei*. Environ Toxicol Chem.

