# Supplementary material for: Predicting chemical bioavailability using microarray gene expression data and regression modeling: A tale of three explosive compounds
Source: BMC Genomics. 2016 Mar 8;17:205. doi: 10.1186/s12864-016-2541-5 (PMC4784335; doi:10.1186/s12864-016-2541-5)
Supplement: Additional file 1: — Description of 18 regression models and their references. (DOCX 21 kb) [file 12864_2016_2541_MOESM1_ESM.docx]

**Regression Methods and References**

The following 18 regression methods were used in the study:

1. Multilinear: Matlab built-in function.
2. Robust linear: Matlab built-in function.
   1. Paper reference: McKean, Joseph W. (2004). "Robust Analysis of Linear Models". *Statistical Science* 19 (4): 562–570.
3. Ridge linear: Matlab built-in function.
   1. Paper reference: [Tikhonov, Andrey Nikolayevich](http://en.wikipedia.org/wiki/Andrey_Nikolayevich_Tychonoff) (1943). "Об устойчивости обратных задач [On the stability of inverse problems]". *[Doklady Akademii Nauk](http://en.wikipedia.org/wiki/Doklady_Akademii_Nauk_SSSR)* [SSSR](http://en.wikipedia.org/wiki/Doklady_Akademii_Nauk_SSSR) **39** (5): 195–198.
4. LASSO regularization linear: Matlab built-in function, LASSO = least absolute shrinkage and selection operator
   1. Paper reference: J. Wolberg (2005). *Data Analysis Using the Method of Least Squares: Extracting the Most Information from Experiments*. Springer. [ISBN](http://en.wikipedia.org/wiki/International_Standard_Book_Number) [3540256741](http://en.wikipedia.org/wiki/Special:BookSources/3540256741).
   2. Paper reference: Friedman, Jerome; Hastie, Trevor; Tibshirani, Rob (2010). “Regularization Paths for Generalized Linear Models via Coordinate Descent”. Journal of Statistical Software, 33: 1-22.
5. Elastic net regularization linear: Matlab built-in function
   1. Paper reference: Zou, Hui; Hastie, Trevor (2005). ["Regularization and Variable Selection via the Elastic Net"](http://citeseerx.ist.psu.edu/viewdoc/summary?doi=10.1.1.124.4696). [*Journal of the Royal Statistical Society*](http://en.wikipedia.org/wiki/Journal_of_the_Royal_Statistical_Society)*, Series B*: 301–320.
   2. Paper reference: Friedman, Jerome; Hastie, Trevor; Tibshirani, Rob (2010). “Regularization Paths for Generalized Linear Models via Coordinate Descent”. Journal of Statistical Software, 33: 1-22.
6. Support Vector Regression (SVR) linear: use libsvm package
   1. Paper reference: Drucker, Harris; Burges, Christopher J. C.; Kaufman, Linda; Smola, Alexander J.; and Vapnik, Vladimir N. (1997); "Support Vector Regression Machines", in *Advances in Neural Information Processing Systems 9, NIPS 1996*, 155–161, MIT Press.
   2. Package reference: <http://www.csie.ntu.edu.tw/~cjlin/libsvm/>
   3. C.-C. Chang and C.-J. Lin. LIBSVM : a library for support vector machines. ACM Transactions on Intelligent Systems and Technology, 2:27:1--27:27, 2011.
7. Stepwise regression: Matlab built-in function
   1. Paper reference: Hocking, R. R. (1976) "The Analysis and Selection of Variables in Linear Regression," *Biometrics, 32.*
8. Ridge 2-degree polynomial (Ridge Poly): use matlab.
9. Ridge exponential (Ridge Exp): use matlab
10. Ridge Gaussian kernel: Written by Ambarish Jash. Code is covered by BSD license.
    1. Package reference: http://www.mathworks.com/matlabcentral/fileexchange/27248-kernel-ridge-regression.
11. SVR 2-degree polynomial (SVR Poly): use libsvm
12. SVR Gaussian kernel: use libsvm
13. SVR Sigmoid kernel: use libsvm
14. Nadaraya-Watson kernel regression: Written by Yi Cao. Code is covered by BSD license.
    1. Paper reference: Nadaraya, E. A. (1964). "On Estimating Regression". *Theory of Probability and its Applications* 9 (1): 141–142.
    2. Package reference: http://www.mathworks.com/matlabcentral/fileexchange/19195.
15. Inverse regression: Matlab built-in function
    1. Paper reference: Dobson, A. J. *An Introduction to Generalized Linear Models*. 1990, CRC Press.
16. Loglog regression: Matlab built-in function.
    1. Paper reference: Dobson, A. J. *An Introduction to Generalized Linear Models*. 1990, CRC Press. (Same as Inverse regression)
17. Regression tree: Matlab built-in function.
    1. Paper reference:  Breiman, Leo; Friedman, J. H., Olshen, R. A., & Stone, C. J. (1984). *Classification and regression trees*. Monterey, CA: Wadsworth & Brooks/Cole Advanced Books & Software.
18. Random Forest regression: Written by Leo. Code is covered by BSD license.
    1. Paper reference:  [Breiman, Leo](http://en.wikipedia.org/wiki/Leo_Breiman) (2001). "Random Forests". [*Machine Learning*](http://en.wikipedia.org/wiki/Machine_Learning_(journal)) 4**5** (1): 5–32.
    2. Package reference: http://www.mathworks.com/matlabcentral/fileexchange/31036-random-forest
